# Construction of 2D/2D Mesoporous WO_3_/CeO_2_ Laminated Heterojunctions for Optimized Photocatalytic Performance

**DOI:** 10.3390/nano13111798

**Published:** 2023-06-04

**Authors:** Wenjie Wang, Decai Yang, Yifan Mou, Lijun Liao, Shijie Wang, Liping Guo, Xuepeng Wang, Zhenzi Li, Wei Zhou

**Affiliations:** Shandong Provincial Key Laboratory of Molecular Engineering, School of Chemistry and Chemical Engineering, Qilu University of Technology (Shandong Academy of Sciences), Jinan 250353, China; 10431200349@stu.qlu.edu.cn (W.W.); wsj0924@qlu.edu.cn (S.W.); wxpchem@hotmail.com (X.W.); wzhou@qlu.edu.cn (W.Z.)

**Keywords:** photocatalysis, WO_3_ nanoplate, mesoporous CeO_2_ nanosheet, Z-scheme, laminated heterojunction

## Abstract

Photocatalytic elimination of antibiotics from the environment and drinking water is of great significance for human health. However, the efficiency of photoremoval of antibiotics such as tetracycline is severely limited by the prompt recombination of electron holes and slow charge migration efficacy. Fabrication of low-dimensional heterojunction composites is an efficient method for shortening charge carrier migration distance and enhancing charge transfer efficiency. Herein, 2D/2D mesoporous WO_3_/CeO_2_ laminated Z-scheme heterojunctions were successfully prepared using a two-step hydrothermal process. The mesoporous structure of the composites was proved by nitrogen sorption isotherms, in which sorption-desorption hysteresis was observed. The intimate contact and charge transfer mechanism between WO_3_ nanoplates and CeO_2_ nanosheets was investigated using high-resolution transmission electron microscopy and X-ray photoelectron spectroscopy measurements, respectively. Photocatalytic tetracycline degradation efficiency was noticeably promoted by the formation of 2D/2D laminated heterojunctions. The improved photocatalytic activity could be attributed to the formation of Z-scheme laminated heterostructure and 2D morphology favoring spatial charge separation, confirmed by various characterizations. The optimized 5WO_3_/CeO_2_ (5 wt.% WO_3_) composites can degrade more than 99% of tetracycline in 80 min, achieving a peak TC photodegradation efficiency of 0.0482 min^−1^, which is approximately 3.4 times that of pristine CeO_2_. A Z-scheme mechanism is proposed for photocatalytic tetracycline by from WO_3_/CeO_2_ Z-scheme laminated heterojunctions based on the experimental results.

## 1. Introduction

Excessive current use of antibiotics has led to the wide distribution of antibiotic residues in the global environment, even in drinking water, which adversely affects human health [1,2]. Thus, the removal of antibiotics is of great importance for life on earth and the sustainable development of human society. Various technologies such as physical purification, chemical degradation, biological degradation, and catalytic elimination have been developed to remove antibiotics from polluted water [3,4]. Among these methods, photocatalysis is one of the most promising routes for residual antibiotic elimination because it is a solar-driven process that does not consume energy [5]. However, the application of photocatalytic antibiotic removal in natural conditions is hindered by the low efficiency of the photocatalytic process. Therefore, research on improving photocatalytic efficiency is significant for antibiotic removal.

Semiconductor-based photocatalytic technology can create electrons and holes under light illumination, which in turn produce superoxide radicals (O_2_^−^) and hydroxyl radicals (·OH) in the presence of water and oxygen [6]. These reactive oxygen radicals and photogenerated holes are capable of oxidizing antibiotics into CO_2_ and H_2_O [7]. Various types of semiconductors, including TiO_2_-based materials [8], bismuth-based photocatalysts [9], g-C_3_N_4_-based systems [10], heterojunction and surface plasmon resonance enhanced photocatalysts [11], and other materials have been explored for photocatalytic removal of antibiotics [12]. Among these photocatalysts, WO_3_ has drawn much attention for visible light pollutant removal because of its narrow band gap [13]. Kim et al. investigated the influence of different inorganic oxidants on the photocatalytic performance of WO_3_ for visible light degradation of 4-chlorophenol, demonstrating charge separation enhancement by oxyanions [14]. Nguyen et al. reported that amoxicillin could be degraded by WO_3_ under simulated solar irradiation, which followed pseudo-first-order kinetics [15]. In addition, near-infrared light photocatalytic removal of methylene blue was achieved on a longitudinally grown WO_3_ nanoparticle surface [16]. However, the photocatalytic activity of WO_3_ is severely restricted by the prompt charge recombination and the lack of superoxide radicals because of its low conduction band position [17]. These drawbacks can be overcome by the formation of heterojunctions with metals or other semiconductors with high conduction band levels. The electron–hole pair separation and visible light absorption were much improved by the loading of Ag on WO_3_ surfaces, producing excellent sulfanilamide degradation activity [18]. The formation of WO_3_/carbon nanotubes was also reported to effectively suppress the recombination of charge carriers and promote sonophotocatalytic tetracycline removal efficiency [19]. Oxidizing species were proved to be OH and h^+^ without O_2_^−^ in that research [19].

CeO_2_ is an attractive candidate for constructing heterojunction photocatalysts with other semiconductors because it is chemically stable, nontoxic, and has proper band positions, where the conduction band is positioned at ca. −0.78 eV (vs. NHE) [19]. Oxygen vacancies were proved to be present on the CeO_2_ surface, thereby enhancing charge separation and transfer efficiency [20]. Recently, various heterojunctions such as SnIn_4_S_8_/CeO_2_ [21], Ni/CeO_2_ [22], CdS/CeO_2_ [23], and 3D/0D Cu_3_SnS_4_/CeO_2_ heterojunctions [24] have been reported to show significant improvement in photocatalytic performance. Bahadoran et al. reported an S-scheme WO_3_/CeO_2_ heterojunction with enhanced visible light photocatalytic sulfamerazine degradation performance [25]. Moreover, the photodegradation activity of indigo carmine dye was much improved by the formation of type II WO_3_/CeO_2_ heterojunction nanoparticles [26].

Because of its wide band gap of ca. 3.2 eV, CeO_2_ photocatalysts can only be activated by UV irradiation, but possess decent photocatalytic activity for pollutant degradation with the generation of oxidizing radicals including O_2_^−^ and OH [26]. In contrast, WO_3_ has a narrow band gap of 2.4–2.8 eV, thus exhibiting potential applications in visible-light-driven photocatalytic reactions [27,28]. However, the photogenerated electrons and holes on the WO_3_ surface demonstrate insufficient redox ability due to its band positions [28]. Therefore, the addition of small amounts of WO_3_ to CeO_2_ photocatalysts could extend the light response range from ultraviolet to the visible light region without significant deactivation. Although type II heterojunctions have been investigated for indigo carmine dye removal [26], to our knowledge, the construction of 2D/2D mesoporous WO_3_/CeO_2_ Z-scheme laminated heterojunctions with promoted photocatalytic tetracycline degradation activity has never been reported. We fabricated 2D/2D mesoporous WO_3_/CeO_2_ Z-scheme laminated heterojunctions via a facile two-step hydrothermal process. The transfer efficiency of the photoinduced charges was significantly enhanced by the low-dimension morphology and the formation of the Z-scheme laminated heterostructure. The improved charge transfer efficiency and suppressed electron hole recombination were confirmed by various characterizations, including electrochemical impedance spectra, transient photocurrent responses, surface photovoltage spectroscopy, and photoluminescence.

## 2. Experimental

### 2.1. Synthesis of Mesoporous CeO_2_ Nanosheets, WO_3_ Nanoplates, and WO_3_/CeO_2_ Composites

**Scheme 1:**
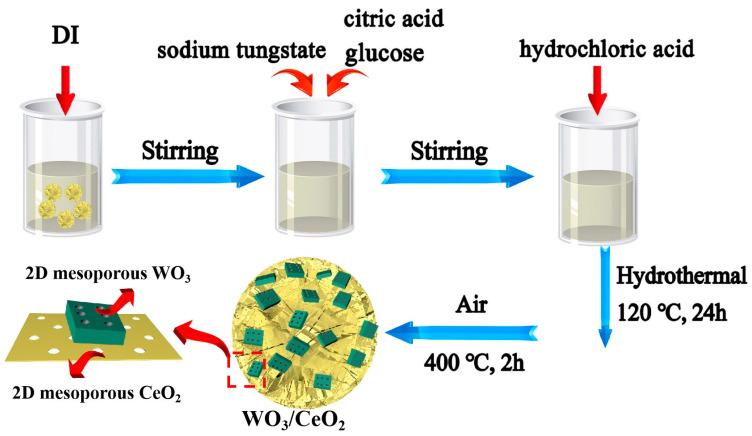
Schematic illustration of the synthesis of 2D/2D mesoporous WO_3_/CeO_2_ laminated heterojunctions.

The 2D CeO_2_ nanosheets were synthesized according to our previously reported method [29]. Typically, 10 mmol glucose was added to 80 mL deionized water and magnetically stirred for 30 min. Subsequently, 15 mmol C_3_H_5_NO and 5 mmol Ce(NO_3_)_3_·6H_2_O were dissolved in the above solution under magnetic stirring for 1 h, and 3 mL NH_3_·H_2_O (28 wt%) was added drop by drop to the mixture and stirred for 5 h, creating a dark brown gel mixture. The mixture was transferred into a Teflon-lined autoclave and maintained at 180 °C for 72 h. The solid products were collected and washed via centrifugation with water and ethanol after reaction. CeO_2_ nanosheets were obtained via thermal treatment at 600 °C for 6 h under a nitrogen atmosphere and subsequent calcination at 400 °C for 4 h under an air atmosphere. The 2D WO_3_ nanoplates were fabricated using a slightly modified procedure [30]. In a typical procedure, 0.06 g Na_2_WO_4_·2H_2_O was first added to 30 mL water. Subsequently, 0.013 g C_6_H_8_O_7_·H_2_O and 0.04 g C_6_H_12_O_6_ were added to the above solution. After 20 min magnetic stirring, 3 mL HCl (6M) was added dropwise to the mixture, and the mixture was transferred to a 50 mL autoclave and heated at 120 °C for 24 h. After the reaction, the product was collected and washed with anhydrous ethanol and deionized water several times by centrifugation. The sample was then heated to 400 °C at a heating rate of 5 °C min^−1^ and maintained for 2 h to obtain mesoporous WO_3_ nanoplates. Mesoporous WO_3_/CeO_2_ heterojunctions were prepared by a two-step hydrothermal method. The synthetic procedure of WO_3_/CeO_2_ is presented in Scheme 1. First, 0.06 g Na_2_WO_4_·2H_2_O and 2.3 g prepared CeO_2_ were added to 30 mL water to form a transparent solution. Subsequently, 0.013 g C_6_H_8_O_7_·H_2_O and 0.04 g C_6_H_12_O_6_ were added to the above solution. After 20 min magnetic stirring, 3 mL HCl (6M) was added drop by drop to the mixture, and the solution was transferred to a 50 mL autoclave and maintained at 120 °C for 24 h. After the reaction, the product was centrifuged and washed with anhydrous ethanol and deionized water several times to remove surface impurities. The sample was then heated to 400 °C at a heating rate of 5 °C min^−1^ and kept for 2 h to improve the crystallinity. A yellow powder was obtained, denoted as xWO_3_/CeO_2_ (x%: mass ratio for WO_3_). The 3WO_3_/CeO_2_, 5WO_3_/CeO_2_, and 7WO_3_/CeO_2_ heterojunctions were obtained using 1.39 g, 2.3 g, and 3.25 g CeO_2,_ respectively, during the synthetic procedure.

### 2.2. Characterizations

X-ray diffraction (XRD) measurements were performed on a Bruker D8 Advance diffractometer (Bruker, Karlsruhe, Germany) using Cu Kα radiation (λ = 1.5406 Å, 40 kV, 40 mA). The step size and scan range were 0.02°, and 5–80°, respectively. The UV-vis absorption spectra were obtained using a UV-visible spectrophotometer (Shimadzu UV-2550, Shimadzu, Tokyo, Japan). A PerkinElmer Lambda 950 UV-vis spectrophotometer (PerkinElmer, Waltham, MA, USA) was used to measure the diffuse reflectance spectrum (DRS) in the wavelength range of 200–1800 nm. Materials morphology was analyzed by scanning electron microscopy (SEM) (Hitachi S-4800, Hitachi, Tokyo, Japan) and transmission electron microscopy (TEM) (JEOLJEM-2100, Jeol, Tokyo, Japan). X-ray photoelectron spectroscopy (XPS) measurements were conducted on a Kratos-Axis ULTRA DLD electron spectrometer with an AlKα (1253.6 eV) excitation source. The results were calibrated using the standard C 1s peak (284.6 eV). Nitrogen sorption measurements were performed with a TriStar 3020 ⅱ automatic analyzer. The Brunaeur-Emmet-Teller (BET) equation and the adsorption branch of BET isotherms were used to obtain the specific surface area and pore size distribution, respectively. The surface photovoltage spectroscopy (SPS) was tested on a homemade instrument in the wavelength range of 200–600 nm. The photoluminescence spectra (PL) were measured using a PELS 55 instrument with a selected excitation wavelength of 275 nm. The photoelectric characteristics of the samples were analyzed on a Princeton VersaSTAT workstation (Princeton Applied Research, Oak Ridge, TN, USA) with a three-electrode system, using a catalyst as the photoanode, platinum as the cathode, Ag/AgCl as the reference electrode, and 0.5 M Na_2_SO_4_ solution as the electrolyte, respectively.

### 2.3. Photocatalytic Activity Measurements

The photocatalytic activity of CeO_2_ and WO_3_/CeO_2_ were measured in the tetracycline (TC) degradation process. First, 100 mg of CeO_2_ or WO_3_/CeO_2_ was added to 100 mL TC solution (10 mg L^−1^). The suspension was kept in the dark for 20 min and analyzed to evaluate the adsorption properties of the sample. Subsequently, the mixture was illuminated under a 300 W Xe lamp (PerfectLight, Beijing, China) with an AM 1.5G filter. The suspension was sampled every 20 min, 5 times in total. The TC concentration was determined by UV-vis spectroscopy at the wavelength of 357 nm.

## 3. Results and Discussion

### 3.1. Morphology and Microstructure

**Figure 1:**
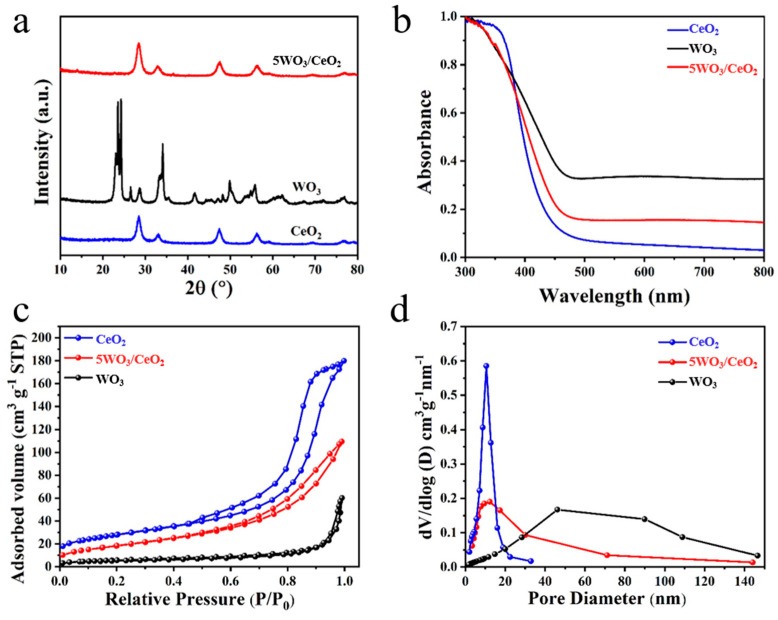
(**a**) XRD patterns, (**b**) UV-vis diffuse reflectance spectra, (**c**) N_2_ sorption isotherms, and (**d**) BJH pore size distribution plots of WO_3_, CeO_2,_ and 5WO_3_/CeO_2_.

The crystal structures of CeO_2_ and WO_3_/CeO_2_ were analyzed by X-ray diffraction (XRD). Figure 1a and Figure S1 show the XRD profiles of the CeO_2_ and WO_3_/CeO_2_ heterojunction photocatalysts. The characteristic peaks of CeO_2_ (JCPDS No. 34-0394) and WO_3_ (JCPDS No. 20-1323) can be identified in the samples of CeO_2_ and WO_3_ [31,32]. The characteristic peaks of WO_3_ are not observed in the XRD patterns of 3WO_3_/CeO_2_ and 5WO_3_/CeO_2_ because the loading amounts of WO_3_ are too low to be detected. The characteristic peaks of both CeO_2_ and WO_3_ can be identified in the XRD pattern of 7WO_3_/CeO_2_, indicating the successful formation of WO_3_/CeO_2_ composites. The subsequent evaluation of photocatalytic TC degradation performance revealed that 5WO_3_/CeO_2_ (5 wt%) demonstrated the best photocatalytic activity. Thus, 5WO_3_/CeO_2_ was used for all the characterizations. The light absorption properties of the as-made samples were analyzed by UV-vis spectroscopy, as shown in Figure 1b. CeO_2_ exhibited strong absorption in the ultraviolet range (200–400 nm) but limited visible light absorption ability. WO_3_ showed strong absorption in the visible light range, thereby enhancing the light absorption ability of the WO_3_/CeO_2_ heterojunctions. Figure S2 presents the UV-vis diffuse reflection spectra of the samples with different WO_3_ contents. The visible light absorption ability of WO_3_/CeO_2_ increases with the WO_3_ content. Figure 1c,d describes the N_2_ sorption isotherms and the corresponding BJH pore size distribution plots of CeO_2_, WO_3_, and WO_3_/CeO_2_. The BET surface area, average pore size, and total pore volume of the samples are presented in Table S1. Typical type IV hysteresis loops can be observed in all three samples, indicating that mesopores are formed in the synthesized samples, which is consistent with the average pore size data. Materials with mesoporous pores offer enhanced accessibility and superior mass transfer [33,34], thus having the possibility to enhance the photocatalytic redox reaction. The average crystallite diameters (D) of the as-prepared samples were obtained by the Scherrer equation [35]:

(1) $$D=\frac{0.89\lambda }{\beta cos\;\theta }$$

*λ*, *β*, and *θ* are the X-ray wavelength, the full width at half maximum of the peak, and the Bragg’s diffraction angle of the peak, respectively. The crystallite size, surface area, pore volume, and main pore size are presented in Table 1.

**Figure 2:**
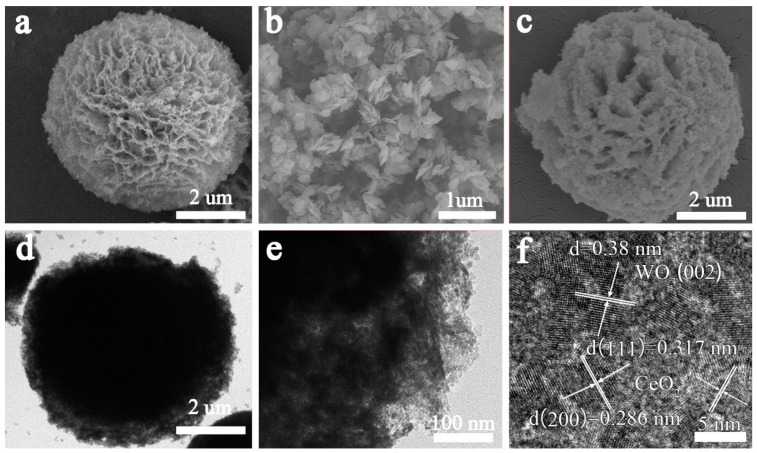
SEM images of CeO_2_ (**a**), WO_3_ (**b**), and WO_3_/CeO_2_ (**c**), and TEM images (**d**,**e**) and HRTEM image (**f**) of WO_3_/CeO_2_.

The morphology and microscopic structure of WO_3_, CeO_2_, and WO_3_/CeO_2_ were studied by scanning electron microscopy (SEM) and transmission electron microscopy (TEM), as demonstrated in Figure 2. The CeO_2_ microspheres (diameter: ca. 4.5 μm) consist of uniform CeO_2_ nanosheets, providing a 2D platform for the formation of laminated heterojunctions. WO_3_ nanoplates are uniformly dispersed on the surface of CeO_2_ nanosheets after combination with CeO_2_ under hydrothermal conditions. Obvious lattice fringes of CeO_2_ and WO_3_ can be identified by a high-resolution TEM image (Figure 2f), indicating the high crystallinity of the formed WO_3_/CeO_2_ composites. The spacing of 0.38 nm is ascribed to the (002) plane of WO_3_, while the lattice fringes of 0.317 nm and 0.286 nm are attributed to the (111) plane and (200) plane of CeO_2_, respectively. The microstructure of the WO_3_/CeO_2_ composites revealed by TEM is in good agreement with the XRD data. In addition, the high-resolution image of WO_3_/CeO_2_ shows that the lattice fringes of WO_3_ and CeO_2_ are in close contact with each other, suggesting the successful construction of heterojunction photocatalysts.

**Figure 3:**
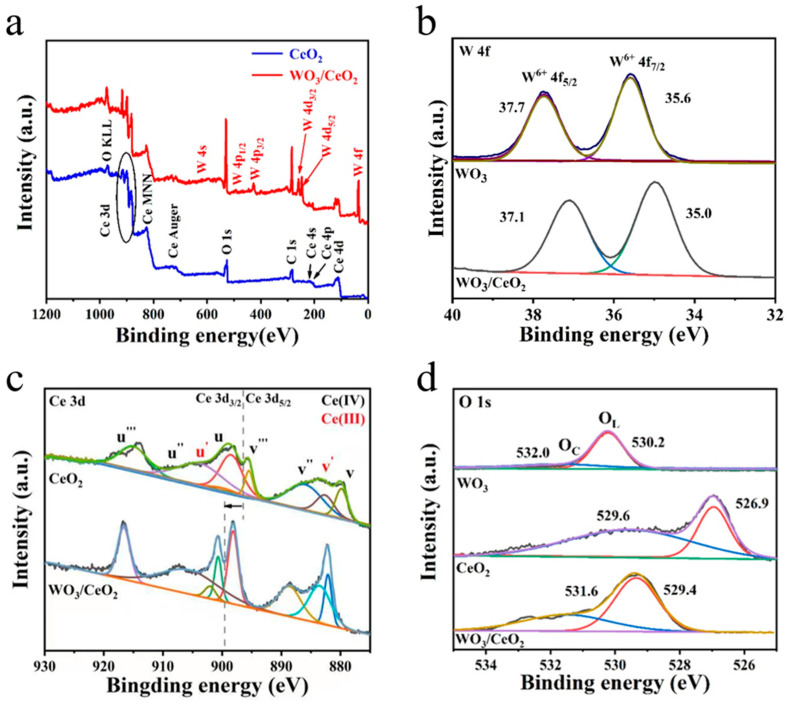
The XPS spectra of WO_3_, CeO_2_ and WO_3_/CeO_2_: (**a**) survey spectra, (**b**) W 4f, (**c**) Ce 3d, (**d**) O 1s.

Figure 3 presents the XPS spectra of CeO_2_ and WO_3_/CeO_2_ composites. All the binding energy positions in the XPS measurements were calibrated using C 1s as the reference at 284.8 eV. The full XPS survey spectra of CeO_2_ and WO_3_/CeO_2_ composites are illustrated in Figure 3a, while the analysis of the high-resolution XPS spectra for W, Ce, and O are presented in Figure 3b–d. The presence of W, O, and Ce elements in the XPS survey spectrum of WO_3_/CeO_2_ provides evidence that the WO_3_/CeO_2_ composites were successfully constructed without impurities. The binding energy at 35.7 and 37.8 eV can be ascribed to W 4f_7/2_ and W 4f_5/2_, confirming the presence of W^6+^ in the WO_3_/CeO_2_ composites [36]. Upon the formation of the WO_3_/CeO_2_ composites, the above two peaks of W 4f shifted to lower energy levels, indicating electron transfer occurs because of the energy level difference. Thus, the electron density on the surface of WO_3_ in the composites increased. As shown in Figure 3c, two kinds of spin-orbit interaction, including Ce 3d_3/2_ (XPS peaks are denoted as u or u-derivatives) and Ce 3d_5/2_ (XPS peaks are denoted as v or v-derivatives), can be clearly observed in the high-resolution Ce 3d XPS spectra. The peaks located at 901.1 eV (u), 903.7 eV (u’), 907.3 eV (u’’), and 916.7 eV (u’’’) can be attributed to Ce 3d_3/2_, while the binding energies at 882.5 eV (v), 884.8 eV (v’), 888.8 eV (v’’), and 898.2 eV (v’’’) are assigned to Ce 3d_5/2_. The O 1s peaks at 529.4 and 531.3 eV originate from lattice oxygen (O_L_: W-O, Ce-O) and chemisorbed oxygen (O_C_), respectively, in WO_3_ and CeO_2_, confirming the firm adhesion between WO_3_ and CeO_2_ in the heterojunction composites [25,29]. Moreover, the Ce 3d, and O 1s main peaks of the WO_3_/CeO_2_ composites demonstrate migration after the construction of composites, suggesting the change of chemical environment and the existence of strong interaction between WO_3_ and CeO_2_.

**Figure 4:**
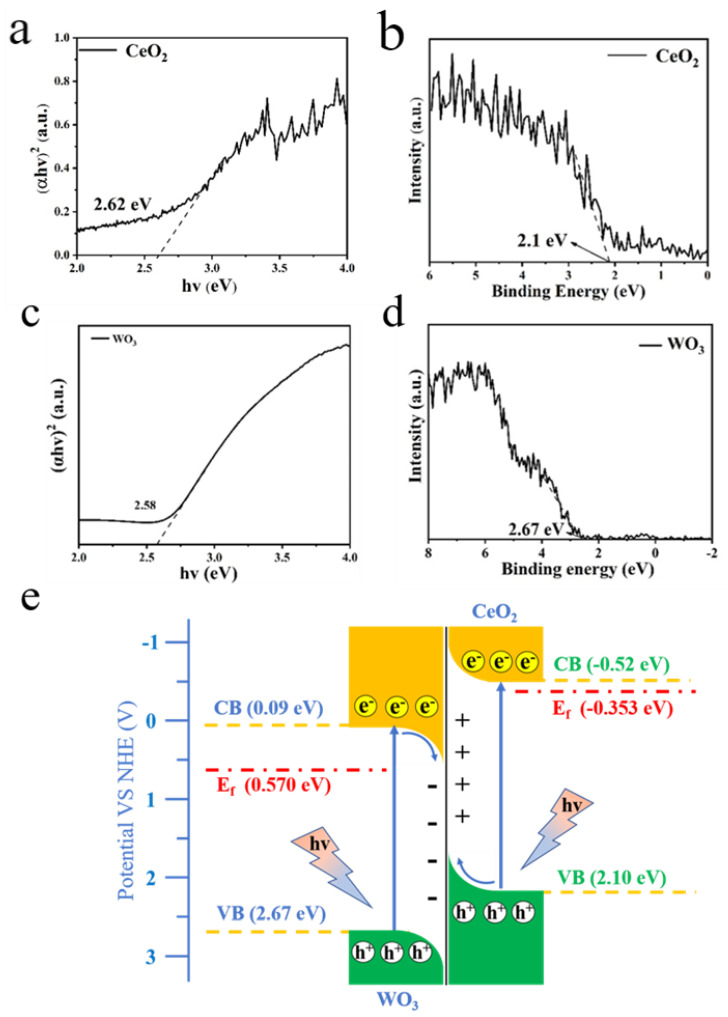
Band gap energies (**a**,**c**) and valance band XPS spectra (**b**,**d**) and band alignments (**e**) of WO_3_ and CeO_2_, respectively.

The energy band gaps (E_g_) for WO_3_ and CeO_2_ were obtained by using the Kubella-Munk formula (αhv = A(hv − E_g_)^η/2^), where A, α, h, and η, are the constant, the absorption coefficient close to the absorption edge, the Planck constant, and the coefficient of the semiconductor, respectively. The value of coefficient η of CeO_2_ and WO_3_ is 4, since both semiconductors have indirect band gaps [37,38]. The E_g_ values of the as-synthesized CeO_2_ and WO_3_ samples were determined to be 2.62 eV and 2.58 eV, respectively, by the Kubella-Munk formula plots derived from the UV-Vis spectra, as presented in Figure 4a,c. The valence band (VB) maximum positions of the materials were normally measured by the valence band XPS spectra [39]. As displayed in Figure 4b,d, the surface valence band edges of CeO_2_ and WO_3_ were estimated to be 2.1 eV and 2.67 eV, respectively, by drawing the tangent line [40]. Based on the empirical equation E_CB_ = E_VB_ − E_g_ (E_CB_, E_VB_, and E_g_ are the conduction band, valence band positions, and the band gap of semiconductors, respectively), and the results of the obtained band gaps and valence band maximum positions, the conduction band (CB) minimum positions of CeO_2_ and WO_3_ are calculated to be −0.52 eV and 0.09 eV, respectively. The energy scale is normally demonstrated in volts using the normal hydrogen electrode (NHE) as a reference [41]. The Fermi levels of WO_3_ and CeO_2_ were reported to be 0.57 and −0.353 eV, respectively [42,43]. It should be noted that the band bending direction in the interface of composites could be significantly influenced by the Fermi level of the two semiconductors [43]. The charge transfer process of WO_3_/CeO_2_ heterojunction is assumed to be a Z-scheme mechanism, which is proved and analyzed below. Therefore, the band edge of CeO_2_ with a high Fermi level is bent upward while the band edge of WO_3_ with a low Fermi level is bent downward, because the electrons migrate from the high Fermi level to the low Fermi level [43]. A built-in electric field in the heterojunction interface could be formed because of the curved band edges and charge transfer equilibrium between CeO_2_ and WO_3_ [44]. Thus, the WO_3_/CeO_2_ heterojunction is probably fabricated with a staggered band alignment, as shown in Figure 4e.

### 3.2. Photocatalytic Activity

**Figure 5:**
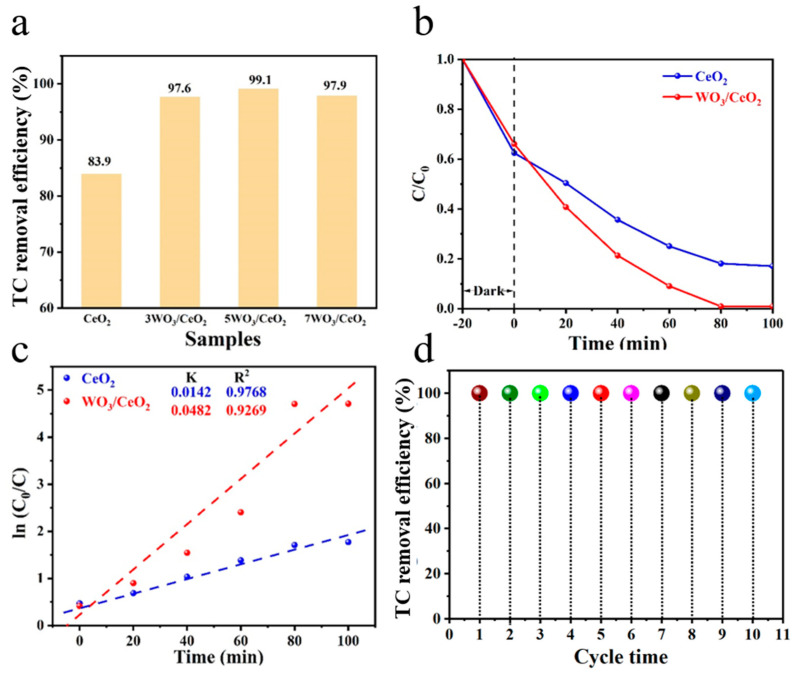
(**a**) TC removal efficiency of the samples with different WO_3_ contents, (**b**) photocatalytic TC degradation performance, (**c**) linear transformation ln(C_0_/C) of the kinetic curves of photocatalytic TC degradation for CeO_2_ and WO_3_/CeO_2_, and (**d**) removal efficiency in cycling tests of photocatalytic TC degradation under AM 1.5G.

The photocatalytic activity of different samples in the tetracycline degradation process was evaluated under AM 1.5G illumination. Photocatalytic TC removal efficiency in the presence of the heterojunction photocatalysts with different amounts of WO_3_ was measured. As shown in Figure 5a, the photocatalytic TC degradation efficiency of CeO_2_ was much improved after the construction of the heterostructure with WO_3_. The photocatalytic efficiency for TC degradation increases with the amount of WO_3_. Under light illumination, 5WO_3_/CeO_2_ (5 wt.% WO_3_) heterojunctions exhibit the highest photocatalytic efficiency, at 99.1% within 80 min for TC removal. The enhanced photocatalytic efficiency of the heterojunctions compared to pristine WO_3_ or CeO_2_ is mainly attributed to the formation of the laminated heterostructure and the mesoporous structure. The heterojunction interface could inhibit the photogenerated charge recombination rate, thereby facilitating the charge transfer and the generation of oxidizing radicals. The mesoporous structure presented a relatively high absorption capacity for tetracycline. Moreover, a 2D structure could shorten the distance of charge transfer in the heterojunction interface. However, 7WO_3_/CeO_2_ (7 wt.% WO_3_) composites showed a decreased TC removal efficiency (97.9% within 80 min) under identical reaction conditions. Despite the enhanced charge separation and transfer efficiency at the interface of the heterojunctions, excess amounts of WO_3_ would result in the coverage of active sites of CeO_2_, thus decreasing the photocatalytic activity. Therefore, 5WO_3_/CeO_2_ composites present the proper tradeoff between enhanced charge separation efficiency and active site coverage. The photocatalytic TC removal efficiency of individual WO_3_ has been measured to be 64.9% within 80 min, which is much lower than that of CeO_2_. As shown in Figure 5b, the photocatalytic TC degradation rate of CeO_2_ decreased quickly with reaction time because of the prompt photoinduced electron–hole combination, reaching a final removal efficiency of 83.9%. Owing to the reduced electron–hole recombination rate, 5WO_3_/CeO_2_ composites demonstrated accelerated TC removal efficiency compared to pristine CeO_2_. As illustrated in Figure 5c, the photocatalytic TC degradation rates were well-matched with a pseudo-first-order kinetic model. The kinetic constant (K) of 5WO_3_/CeO_2_ heterojunctions for TC photodegradation achieved 0.0482 min^−1^, which was approximately 3.4 times that of pristine CeO_2_. To verify the outcome of the TC degradation performance, the total organic carbon (TOC) concentration after the reaction in the presence of WO_3_/CeO_2_ was measured using a carbon analyzer (TOC 5000A Shimadzu), as illustrated in Table S2. The TOC concentration after the reaction photocatalyzed by 5WO_3_/CeO_2_ heterojunctions was determined to be 1.6 mg L^−1^, which is in agreement with the UV-Vis spectroscopy measurements.

The reusability of the optimized photocatalysts (5WO_3_/CeO_2_) was evaluated in 10 cycling tests, and the TC photoremoval efficiency is presented in Figure 5d. The heterojunction composites exhibited stable TC elimination efficiency under light illumination, which was almost 100% maintained after ten consecutive cycling measurements, suggesting that the as-synthesized composites possess relatively high photocatalytic stability in the TC elimination process. Table 2 presents the photocatalytic tetracycline degradation performance of 2D/2D mesoporous WO_3_/CeO_2_ compared with reported CeO_2_-based materials. It can be clearly seen that the as-synthesized WO_3_/CeO_2_ laminated heterojunctions exhibit superior photocatalytic TC removal activity compared to other photocatalysts. Moreover, the cycling tests of photocatalytic TC degradation in this study were performed 10 times without apparent photocatalyst deactivation, while only 4 or 5 cycling tests were conducted in the previous studies.

**Figure 6:**
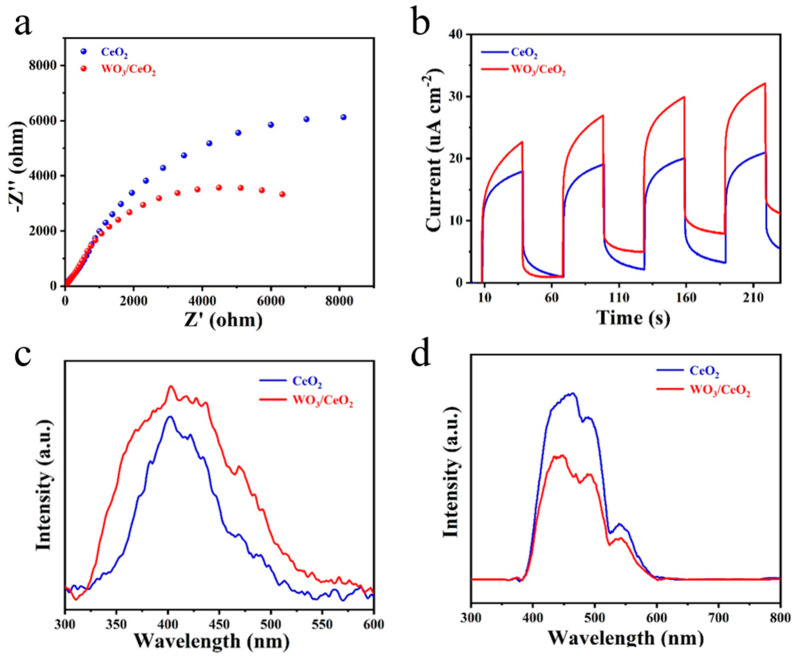
EIS Nyquist plots (**a**), transient photocurrent responses (**b**), surface photovoltage spectroscopy (**c**), and PL spectra (**d**) of CeO_2_ and WO_3_/CeO_2_.

To get insights into the photocatalytic process of TC removal, photoelectrochemical measurements were conducted and presented in Figure 6 to study the separation and transfer efficiency of photoinduced charge carriers on the photocatalyst surface. Figure 6a shows the electrochemical impedance spectra (EIS) measurements of the as-prepared samples, which were conducted at the open-circuit potential of the as-made photocatalysts. The diameter of the obtained semicircle of WO_3_/CeO_2_ composites is clearly smaller than that of the pristine CeO_2_, indicating that composites with heterostructures have lower charge transfer resistance. The transient photocurrent responses of CeO_2_ and WO_3_/CeO_2_ were measured under intermittent light illumination, as illustrated in Figure 6b. The photocurrent density of WO_3_/CeO_2_ was much higher than that of CeO_2_, indicating that the WO_3_/CeO_2_ laminated heterojunction composites exhibited slower charge recombination rate and stronger electron–hole separation ability. In addition, repeatable photocurrent responses in every illumination cycle can be observed, showing the excellent stability of the constructed heterojunctions. Figure 6c presents the surface photovoltage spectroscopy (SPV) data of the samples. The SPV measurements of WO_3_/CeO_2_ demonstrate much-increased surface voltage under light irradiation compared to CeO_2_, showing the accelerated charge transfer efficiency on the heterojunction surface [53]. The suppressed-charge recombination rate was also examined using PL emission measurements, as shown in Figure 6d. The PL spectra of WO_3_/CeO_2_ show clear photoluminescence quenching compared to pure CeO_2_ at an excitation wavelength of 365 nm. This result demonstrates that the efficient suppression of photogenerated electron–hole recombination was achieved via the formation of heterojunction interfaces in WO_3_/CeO_2_ composites. All the above measurements provided solid evidence for the enhanced charge separation and transfer efficiency and suppressed electron–hole recombination, which improves photocatalytic activity for TC degradation.

**Figure 7:**
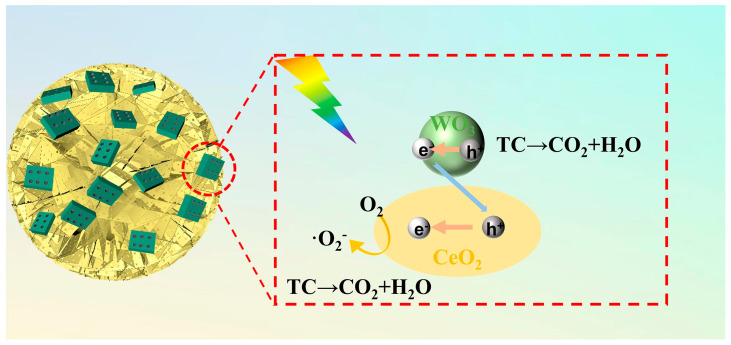
Proposed Z-scheme mechanism for photocatalytic TC degradation on WO_3_/CeO_2_ surface.

All obtained results for TC photodegradation and heterojunction characterizations led to the proposed mechanism for photocatalytic TC elimination on the WO_3_/CeO_2_ surface, as illustrated in Figure 7. As previously mentioned, the band alignment of WO_3_/CeO_2_ is assumed to be staggered according to the measured and calculated band positions. The electrons are excited under light irradiation and migrated from the valence band to the conduction band, thereby leaving holes with positive charges in the valence band. If type II heterojunctions were formed in the as-synthesized WO_3_/CeO_2_ composites, the excited electrons would transfer from the conduction band of CeO_2_ to that of WO_3_. The photogenerated holes migrate from the valence band of WO_3_ to that of CeO_2_. However, the photoinduced electrons in the conduction band of WO_3_ could not reduce oxygen into superoxide radicals because of the more positive CB position of WO_3_ (0.50 eV vs. NHE) compared to the O_2_/·O_2_^−^ potential (−0.33 V vs. NHE) [54]. Likewise, the photogenerated holes in the VB of both WO_3_ (2.62 eV vs. NHE) and CeO_2_ (2.1 eV vs. NHE) could not oxidize H_2_O to form ·OH, due to the more negative VB potential of the two materials compared to the H_2_O/·OH potential (2.72 V vs. NHE) [54]. In that scenario, O_2_^−^ and ·OH cannot be generated in the TC degradation process. Thus, the typical type II heterojunction mechanism was impractical in the WO_3_/CeO_2_ composites. Accordingly, the Z-scheme charge transfer mechanism was applied to the WO_3_/CeO_2_ heterojunction, as shown in Figure 7. Electrons moved from WO_3_ (CB) to CeO_2_ (VB). The electrons accumulated in the CB of CeO_2_ easily oxidize O_2_ into O_2_^−^. Photogenerated holes in WO_3_ (VB) and·O_2_^−^ would degrade TC simultaneously under light irradiation. Both CeO_2_ and WO_3_ are two-dimensional (2D) materials, as illustrated in Figure 2, as 2D photocatalysts have significant advantages in charge-transfer kinetics [55]. Thanks to the 2D morphology and the formation of the Z-scheme laminated heterojunction, the spatial charge separation and transfer efficiency are much enhanced under light illumination. Surface-adsorbed oxygen can capture photogenerated electrons to form superoxide radicals (O_2_^−^). Tetracycline is eventually oxidized into CO_2_ and water by the photoinduced radicals and holes on WO_3_/CeO_2_ surface. However, this mechanism is merely an assumption based on the ·OH and·O_2_^−^ formation mechanism. The detection of reactive species such as ·O_2_^−^ and the TC oxidation products will be performed in further research.

## 4. Conclusions

In summary, 2D/2D mesoporous WO_3_/CeO_2_ photocatalysts were constructed via a facile two-step hydrothermal process. The heterojunction structure was confirmed by high-resolution transmission electron microscopy (HRTEM). The light absorption ability of the composites was improved because of the presence of WO_3_. Based on the measured and calculated band positions and the Fermi level positions, the band alignment of WO_3_/CeO_2_ was assumed to be a staggered Z-scheme heterojunction. The charge separation and migration efficiency were much enhanced in the mesoporous WO_3_/CeO_2_ heterojunction interface, as confirmed by electrochemical impedance spectra, transient photocurrent responses, and surface photovoltage spectroscopy. The suppression of photogenerated charge recombination was proved by photoluminescence. Thanks to the much enhanced charge migration and separation efficiency, the optimized 5WO_3_/CeO_2_ (5 wt.% WO_3_) Z-scheme laminated heterojunction photocatalysts demonstrated tetracycline photodegradation efficiency of 99.1% within 80 min, achieving a peak TC photodegradation efficiency of 0.0482 min^−1^, which is approximately 3.4 times that of pristine CeO_2_. Under light irradiation, the fabricated photocatalysts maintained high TC removal efficiency for 10 cycles. The 2D/2D heterostructure proposed here provides the possibility of rare-earth-metal-based composites for efficient, stable, and reusable water purification.

## Figures and Tables

**Table 1:** Crystallite size, surface area, pore volume, and main pore size of the samples.

Catalyst	*D* (nm)	Surface Area (m^2^ g^−1^)	Pore Volume (cm^3^ g^−1^)	Pore Size (nm)
CeO_2_	19.3	100.3	0.21	9.3
WO_3_	20.1	22.0	0.20	44.7

**Table 2:** Comparison of photocatalytic tetracycline degradation efficiency of 2D/2D mesoporous WO_3_/CeO_2_ with reported values.

Photocatalyst	Time (min)	Removal Efficiency (%)	Number of Cycling Test	Ref.
BiOCl@CeO_2_	120	90	4	[45]
CeO_2_/carbonate doped Bi_2_O_2_CO_3_	90	79.5	4	[46]
ZnO/CeO_2_@Halloysite nanotubes	90	94	4	[47]
SnO/CeO_2_	140	99	5	[48]
AgI/CeO_2_	60	94.34	not studied	[49]
AgBr/CeO_2_	120	77.8	not studied	[50]
CeO_2_/Ti_3_C_2_-MXene	60	80.2	4	[51]
Ag_2_O/AgBr–CeO_2_	60	93.68	4	[52]
2D/2D mesoporous WO_3_/CeO_2_	80	99.1	10	this study

## Data Availability

Data will be made available on request.

## Supplementary Materials

The following are available online at https://www.mdpi.com/article/10.3390/nano13111798/s1, Figure S1: XRD patterns of samples with different WO_3_ contents, Figure S2: UV-vis diffuse reflectance spectra of samples with different WO_3_ contents, Figure S3: Scanning electron microscope of samples with different WO_3_ contents. (a) 3WO_3_/CeO_2_, (b) 5WO_3_/CeO_2_ and (c) 7WO_3_/CeO_2_, Table S1: The structure parameters of WO_3_, CeO_2_ and WO_3_/CeO_2_, Table S2: The total organic carbon (TOC) concentration before and after photocatalytic TC degradation.
