# Supplementary material for: Construction of 2D/2D Mesoporous WO3/CeO2 Laminated Heterojunctions for Optimized Photocatalytic Performance
Source: Nanomaterials (Basel). 2023 Jun 4;13(11):1798. doi: 10.3390/nano13111798 (PMC10254501; doi:10.3390/nano13111798)
Supplement: Supplementary file 1 [file nanomaterials-13-01798-s001.zip › nanomaterials-2426810-supplementary.pdf]

Construction of 2D/2D Mesoporous WO_3/CeO_2 Laminated Heterojunctions Toward Optimized Photocatalytic Performance

Wenjie Wang#, Decai Yang#, Yifan Mou, Lijun Liao*, Shijie Wang, Liping Guo, Xuepeng Wang, Zhenzi Li*, Wei Zhou

Shandong Provincial Key Laboratory of Molecular Engineering, School of Chemistry and Chemical Engineering, Qilu University of Technology (Shandong Academy of Sciences), Jinan 250353, P. R. China

Email: lijun.liao@hotmail.com; zzli@qlu.edu.cn

W. Wang and D. Yang contributed equally to this work.

S1.1 Chemicals

Sodium tungstate ($Na_2WO_4{\cdot}2H_2O$), Glucose ($C_6H_{12}O_6$), acrylamide (C_3H_5NO), cerium nitrate ($Ce(NO_3)_3{\cdot}6H_2O$), and ammonia ($NH_3{\cdot}H_2O$) were purchased from Aladdin Industrial Inc. Ethanol (EtOH), hydrochloric acid (HCl), citric acid ($C_6H_8O_7{\cdot}H_2O$) and potassium hydroxide (KOH) were of analytical grade and purchased from Tianjin Kemiou Chemical Reagent Co., Ltd. All chemicals were utilized without additional treatment.

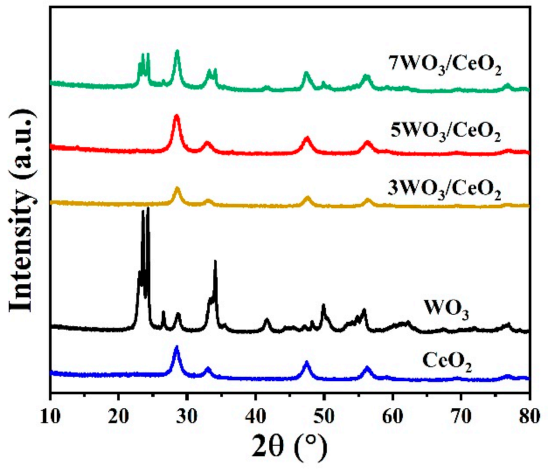

Fig. S1. XRD patterns of samples with different WO_3 contents.

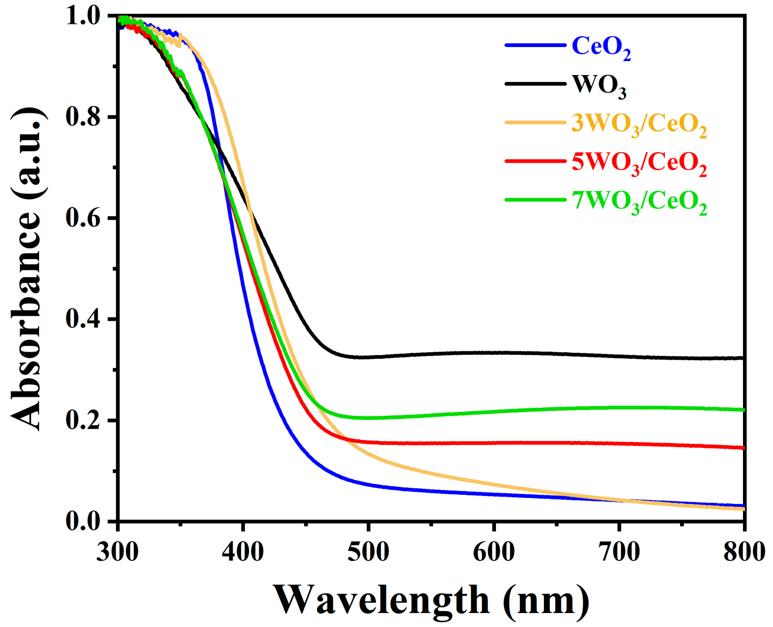

Fig. S2. UV-vis diffuse reflectance spectra of samples with different WO_3 contents.

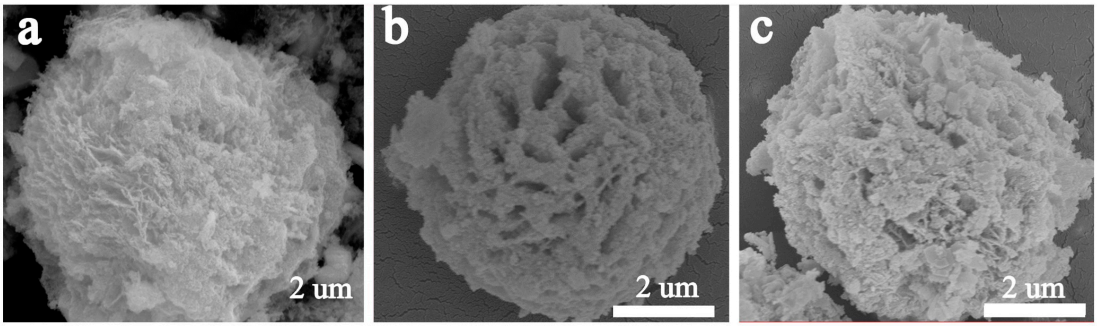

Fig. S3. Scanning electron microscope of samples with different WO_3 contents. (a) $3WO_3/CeO_2$, (b) $5WO_3/CeO_2$ and (c) $7WO_3/CeO_2$.

Table S1. The structure parameters of WO_3 , CeO_2 and WO_3/CeO_2.

Parameters	WO_3	CeO_2	WO_3/CeO_2
BET surface area (m^2 g^{-1})	25	100.3	67.9
Pore size (nm)	46.5	9.3	9.56
Pore volume (cm^3 g^{-1})	0.2	0.21	0.15

Table S2. The total organic carbon (TOC) concentration before and after photocatalytic TC degradation.

Catalysts	TOC concentration before reaction (mg L^{-1})	TOC concentration after reaction (mg L^{-1})
CeO_2	9.8	1.6
$3WO_3/CeO_2$	10.3	0.3
$5WO_3/CeO_2$	10.1	0.1
$7WO_3/CeO_2$	9.9	0.3
